# Comparison of the efficacy between bilateral proximal tubal occlusion and total salpingectomy on ovarian reserve and the cholinergic system: an experimental study

**DOI:** 10.3906/sag-2002-179

**Published:** 2020-06-23

**Authors:** Remzi ATILGAN, Şehmus PALA, Tuncay KULOĞLU, Cengiz ŞANLI, Şeyda YAVUZKIR, Zehra Sema ÖZKAN

**Affiliations:** 1 Department of Obstetrics and Gynecology, School of Medicine, Fırat University, Elazığ Turkey; 2 Department of Histology and Embryology, School of Medicine, Fırat University, Elazığ Turkey; 3 Department of Obstetrics and Gynecology, School of Medicine, Kırıkkale University, Kırıkkale Turkey

**Keywords:** Ovarian reserve, proximal tubal occlusion, CHRM1, rat

## Abstract

**Background and aim:**

To compare the effects of bilateral proximal tubal occlusion and bilateral total salpingectomy on ovarian reserve and the cholinergic system via rat experiment.

**Materials and methods:**

Twenty-one adult female rats were randomly divided into the following three groups:G1 (n = 7), sham group; G2 (n = 7), bilateral total salpingectomy group; and G3 (n = 7), bilateral proximal tubal occlusion group. Four weeks later, the abdomen of the rats was opened. The right ovarian tissues were stored in 10% formaldehyde, whereas the left ovarian tissues were stored at –80 °C in aluminum foil. Serum samples were evaluated for antimullerian hormone. The right ovary was used for histological and immunoreactive examination, and the left ovary was used for tissue MDA analysis. Tissue samples were analyzed for MDA levels with spectrophotometric measurement, apoptosis with TUNEL staining, fibrosis score with Mason trichrome staining, ovarian reserve with HE staining, and cholinergic receptor muscarinic 1 (CHRM1) level with immunoreactivity method.

**Results:**

Compared to G1 and G3, the number of corpus luteum with secondary follicles was significantly lower in G2, whereas the number of ovarian cysts and fibrosis and apoptosis scores increased significantly. The CHRM1 immunoreactivity was significantly lower in G2 than in G1 and G3.

**Conclusions:**

Compared to the bilateral proximal tubal occlusion performed by using bipolar cautery, bilateral total salpingectomy in rats leads to a significant damage in ovarian histopathology and the cholinergic system.

## 1. Introduction 

Hydrosalpinx (HX) has detrimental effects on implantation, pregnancy, live birth, and early pregnancy loss rates due to some effects such as reduction in endometrial receptivity during in vitro fertilization and embryo transfer (IVF–ET) and mechanical washing of the embryo from the uterus [1,2]. In a systematic review and metaanalysis, it was shown that total salpingectomy (TS) increases the rates of implantation, clinical pregnancy rates, and live birth rates without impairing ovarian response during IVF treatment in patients with HX [3]. Surgical interventions that block the communication between the tube and the uterine cavity such as laparoscopic proximal tubal occlusion (PTO) and laparoscopic removal of HX with TS prevent the leakage of HX fluid into the uterine cavity [4]. In a prospective randomized controlled trial, PTO was shown to be a potentially useful surgical procedure that significantly improves the chance of successful implantation and clinical and ongoing pregnancy when performed in women with unilateral or bilateral HX prior to IVF treatment. It was reported that PTO may be considered a viable alternative when salpingectomy is technically difficult or not feasible [5]. 

In a randomized controlled trial, PTO was shown to be superior to salpingectomy as a surgical treatment for patients with HX undergoing IVF–ET in terms of ovarian reserve. However, two surgical techniques were reported to be associated with comparable pregnancy rates [6,7]. However, in the comparisons between the two methods, we saw the lack of ovarian histopathologic examinations. It is ethically very difficult to perform histopathological studies on the ovarian tissue in the reproductive period. Thus, we planned to show the effects of both methods on ovarian tissue in this experimental study. There are increasing concerns regarding TS as it was reported to affect the ovarian reserve because of the possibility of damage to tubal and ovarian vascular structures due to their close proximity [8–13]. However, in a metaanalysis, it was shown that TS does not compromise ovarian reserve in the short term [14]. The ovarian reserve may be less affected because the probability of trauma to the mesosalpinx decreases in PTO, which is an easier and less invasive procedure with fewer postoperative adhesions compared to TS. It has similar success rates with salpingectomy [4,15]. 

In ovaries, systemic and local signaling factors, such as hormones, growth factors, and neurotransmitters determine the fate of follicles [16,17]. Acetylcholine (ACh) is a prominent neurotransmitter because it is synthesized by granulosa cells (GCs), which are the main cellular components of the follicle. The ovarian cholinergic system, consisting of muscarinic receptors, is part of the nonneuronal ACh production and local ACh activity [18,19]. Previous studies have shown the trophic and growth-promoting effects of ACh through the muscarinic receptors in cattle and human ovaries [20].

This study aimed to compare the effects of PTO performed by using bipolar cautery and bilateral TS on ovarian histopathology and cholinergic system in rats. 

## 2. Materials and methods

This experimental study was carried out in the Experimental Animals Laboratory of Fırat University Medical Faculty between September 2017 and January 2018. Twenty-one-week-old adult female Wistar–Albino rats, weighing 190–220 g and having regular cycles, were fed with standard pellet feeds and city water in a room with 21–23 °C of constant temperature and in a photoperiod of 12 h of light (08:00–22:00) and 12 h of dark. This study was approved by the Fırat University Ethical Committee (Date: 16. 02. 2017, Meeting Number: 2017⁄ 04, Decision No: 52) and performed in the Fırat University Experimental Investigations Department in accordance with the international ethical standards on experimental animal studies. All of the authors have a certificate of laboratory animal experiments. 

### 2.1. Experimental protocol

The rats which were found to be in the estrus phase in the vaginal cytology follow-up were randomly divided into the following three groups: in Group (G)1 (n = 7), the abdomen of these rats was opened and closed, and sham operation was performed; in G2 (n = 7), bilateral TS was applied; and in G3 (n = 7), bilateral PTO was performed with bipolar cautery. 

The rats were placed in the supine position on the operating table. Skin of the rats was shaved before surgery. Antiseptic conditions were achieved by applying 10% povidone iodine solution to the operation area. The abdomen of the rats was opened with a midline incision.

#### 2.1.1. TS procedure

The fallopian tube was totally clamped just above the ovarian tissue with the clamp. Under the clamp, the tuba uterina was tied with 5⁄ 0 vicryl suture. After opening of the clamp, the tuba uterina was cut off with the microsurgical scissors just above the binding suture. Thus, bleeding was controlled and cautery was not used.

#### 2.1.2. PTO procedure

The connection of the uterine horn with the ovarian tissue and tuba uterina was determined. Cauterization was performed using bipolar cautery from the junction of the tuba uterine with the uterine horn. Bipolar cauterization with microsurgical cautery (monopolar: 300 W ⁄ 400 Ω and bipolar: 120 W ⁄ 100 Ω, 400 kHz Intl. 10s ⁄ 30s. Profesyonel Elektronik San. ve Tic. AŞ, Ankara/Turkiye) was performed for approximately 3 s (50 W) during which the coagulation was observed with a surgical microscope.

The abdomens of the rats were opened under general anesthesia using ketamine (Ketalar, Eczacibasi Warner - Lambert, Istanbul, Turkey) 75 mg/kg and xylazine hydrochloride (Rompun, Bayer, İstanbul, Türkiye) 10 mg/kg i.m. Four weeks later, the abdomens of the rats were opened again under general anesthesia. The ovaries were evaluated macroscopically. The number and size of macroscopic cysts in the ovaries were determined. Then, the right ovarian tissues were removed and stored in 10% formaldehyde, whereas the left ovarian tissues were wrapped with aluminum foil and frozen and stored at –80 °C until required, and MDA analysis was performed after dissolution. Blood samples taken intracardiacally were centrifuged at 3000 rpm for 10 min to separate the serum, which was then placed into Eppendorf tubes and stored at –80 °C. Decapitation was performed after blood sampling. The antimullerian hormone (AMH) assay was performed using rat AMH ELISA kits (Catalog no: E ‐ EL - R0640; Elabscience Biotechnology; Wuhan, China). 

### 2.2. Tissue sampling

The obtained ovarian tissues were cleaned with cold (+4 °C) 0.9% sodium chloride (NaCI) and dried with tarnishing paper. Then, the tissues were homogenized for 3 min at 16,000 rpm in 0.01-M phosphate buffered saline (PBS) solution (1: 9, w: v) with a homogenizator (Ultra Turrax Type T25- B, IKA Labortechnic, Germany). Homogenization was performed in an ice container. The homogenate was divided into its supernatants by centrifugation at 5000× *g* for 1 h (at +4 °C). The malondialdehyde (MDA) levels in each supernatant were determined with the appropriate methods. 

### 2.3. Determination of the tissue MDA levels

Determination of the MDA levels was based on the coupling of MDA with thiobarbituric acid at +95 °C. Determination of lipid peroxidation depends on the spectrophotometric measurement at 532 nm of the pink complex obtained by the incubation of 0.8% thiobarbituric acid (TBA) with tissue homogenate in boiling water bath for 1 h under aerobic conditions with pH: 3.5. For the measurements, 1,1,3,3 tetraetoxypropan was used as the standard. The results were expressed as nmol/mL.

### 2.4. Histological evaluations

The right ovarian tissues obtained in each group were embedded in paraffin blocks after fixing with 10% formaldehyde. Sections of 4–6 mm thickness were obtained from those paraffin blocks. The sections were stained with Masson’s trichrome dye and hematoxylin and eosin (H&E), and examined and photographed under the microscope. In the calculation of the ovarian reserve, ovarian follicles were defined with the method described by Mazaud [21]. The fibrosis was assessed with Masson’s trichrome staining and scored from 0 to 3 semiquantitatively as follows: 0 = no fibrosis, +1 = low fibrosis, +2 = intermediate fibrosis, +3 = severe fibrosis [22]. 

### 2.5. Terminal deoxynucleotidyl transferase dUTP nick end labelling (TUNEL) staining

Sections of 5–6 µm thickness obtained from paraffin blocks were mounted on polylysine glass slides. Following the instructions by the manufacturer, ApopTagPlus Peroxidase in situ Apoptosis Detection Kit (Chemicon, cat no: S7101, USA) was used to detect the apoptotic cells. Slides were evaluated through microscopic examination (Novel N - 800M). In the evaluation of TUNEL staining, blue-stained nuclei by Harris hematoxylin were evaluated as normal, whereas cells displaying brown-stained nuclei were considered apoptotic. At 10× magnification, at least 500 normal and apoptotic cells were detected in the randomly selected regions of the sections. Apoptotic index (AI) was calculated by taking the ratio of the apoptotic cells to the total (normal + apoptotic) cells [23].

### 2.6. Immunohistochemical examination

Deparaffinized tissues were passed through graded alcohol series and boiled in a citrate buffer solution at pH 6 in a microwave oven (750 W) for 12 min for antigen retrieval. To prevent surface staining, after treating with Ultra V Block (TA – 125- UB, the Lab Vision Corporation, USA) solutions, the tissues were incubated with primary antibodies for 60 min [CHRM1 was purchased from Boster (Cholinergic receptor, muscarinic 1, catalog number: PA 2202, Boster, 3942 B Valley Ave, Pleasanton, CA, 94566)]. After the application of primary antibodies, tissues were incubated with secondary antibodies (30 min) (biotinized anti-mouse/rabbit IgG, Diagnostic BioSystems, KP 50 A, Pleasanton, USA), streptavidin alkaline phosphatase (30 min) (TS – 060- AP, the Lab Vision Corporation, USA), and fast red substrate system (TA – 125- AF, the Lab Vision Corporation, USA). Tissues that were exposed to contrasting staining with Mayer’s hematoxylin were treated with phosphate-buffered saline (PBS) and distilled water, then closed with the appropriate shutdown solution. The prepared tissues were examined and evaluated under the Olympus BX 50 light microscope (Olympus Corporation, Tokyo, Japan) and photographed. Extensity of the staining was taken as the basis when evaluating the immunohistochemical staining. A histoscore was derived from the distribution (0.1 = under 25%; 0.4 = 26–50%; 0.6 = 50–75%; 0.9 = 76–100%) and the intensity (0 = no staining; +0.5 = very little staining; +1 = little staining; +2 = medium staining; +3 = very strong staining) of the staining immunoreactivity (Histoscore = distribution × intensity) [24].

### 2.7. Statistical analysis

When a power analysis of 80% power and 0.05 significance level was performed for the variable with the widest standard deviation from the variables to be used in the study, it was calculated that there should be at least 5, optimally 7 subjects in each group. SPSS 21.0 software (SPSS Inc., Chicago, IL, USA) was used for the statistical analysis of data. The Kolmogorov–Smirnov and Shapiro–Wilk tests were used as tests of normality for the continuous variables. Nonnormally distributed data was expressed as the median (minimum–maximum). The comparison of continuous variables among three groups was done with the Kruskal–Wallis test. Binary comparisons were done with the Mann–Whitney U-test. Categorical variables were compared with chi-squared test and Fisher’s exact test where applicable. P-value smaller than 0.05 was considered statistically significant.

## 3. Results

The experiment was successfully completed in all rats. None of the rats died in the study and all were included in the study.

### 3.1. Ovarian reserve

**Figure 1:**
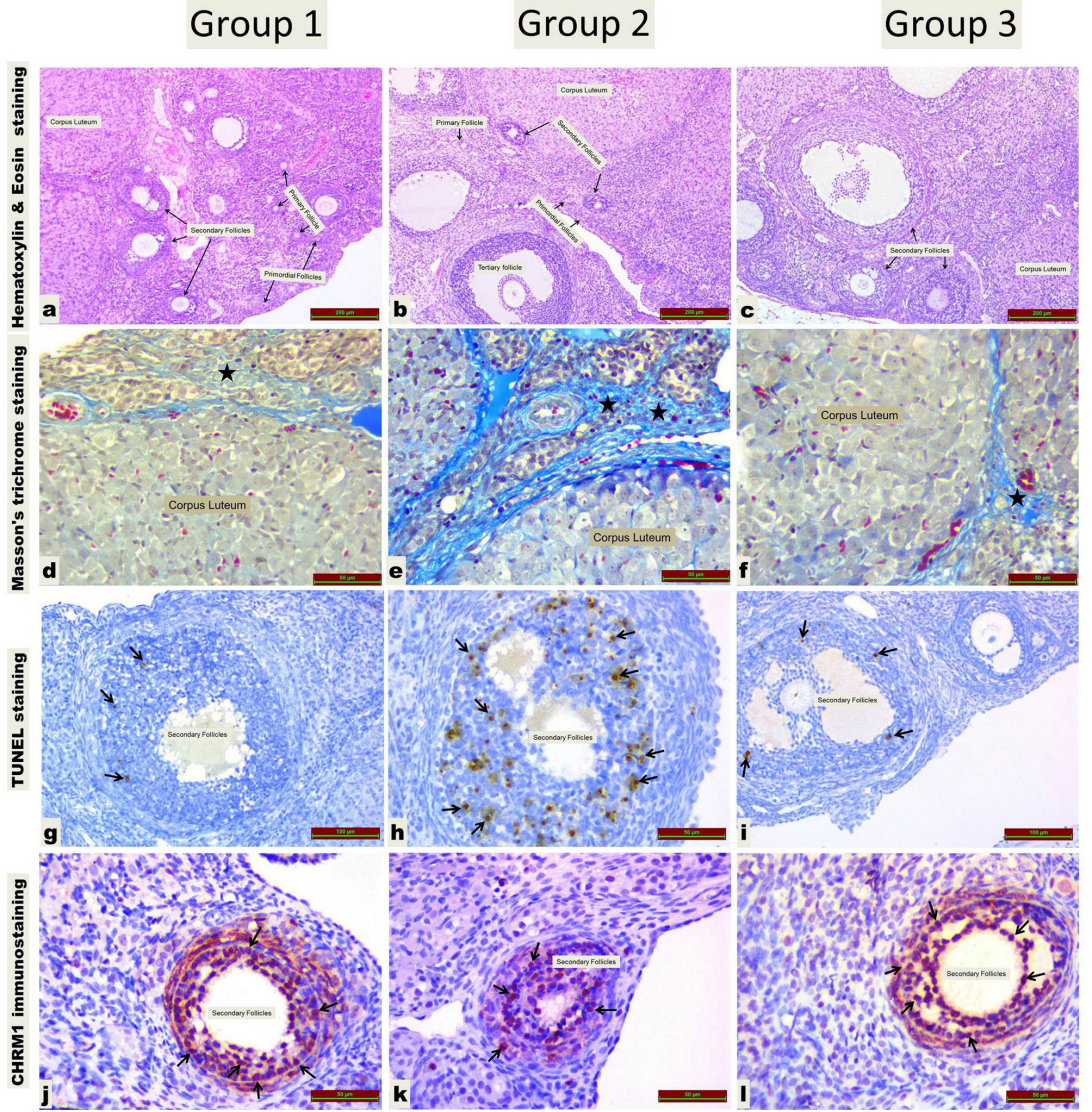
Ovarian follicles, fibrosis, apoptosis, and CHRM1 immunoreactivity in all groups.a,b,c: different types of follicles; d,e,f: fibrosis areas (black star); g,h,i: TUNEL-positive cells (black arrow); j,k,l: chrm 1 immune positivity is observed in granulosa cells of secondary follicle (black arrow).Group 1 = Sham group; Group2 = Total salpingectomy group; Group3 = Proximal tubal occlusion group; CHRM1 = cholinergic receptor muscarinic 1

When G1 and G2 were compared, primordial (P = 0.259) primary (P = 0.165) and tertiary (P = 0.017) follicle numbers were similar. Secondary follicles (P = 0.001), and corpus luteum (CL) (P = 0.001) were significantly decreased in G2 than in G1. There was no macroscopic cyst formation in G1, new ovarian cyst formation with a diameter of 11 (5–15) mm was observed in G2 (P = 0.001), (Table 1), (Figures 1a–1c).

**Table 1:** Table 1. Comparison of cyst formation and ovarian reserve with follicle numbers among groups.

Groups	Primordial follicle	Primary follicle	Secondary follicle	Tertiary follicle	Corpus luteum	Ovarian cyst diameter (mm)/count
G1 (n = 7)	11 (10–13)	12 (9–14)	12 (9–15)	2 (1‐2)	21 (19–24)	0.00 (0–0) ⁄ 0
G2 (n = 7)	12 (9–13)	12 (8–13)	5 (3–6)	0 (0–1)	12 (10–17)	11 (5–15)⁄ 12
G3 (n = 7)	11 (9–15)	13 (9–15)	11 (9–12)	2 (0–2)	15 (13–19)	0.00 (0–0) ⁄ 0
P values	G 1–G2:0.259 G1–G3:0.318 G2–G3:0.165	G1–G2:0.165 G1–G3:0.710 G2–G3:0.128	G1–G2:0.001 G1–G3:0.259 G2–G3:0.001	G1–G2:0.017 G1–G3:0.097 G2–G3:0.535	G1–G2:0.001 G1–G3:0.001 G2–G3:0.017	G1–G2:0.001 G1–G3:1.000 G2–G3:0.001

When G1 and G3 were compared, primordial (P = 0.318), primary (P = 0.710), secondary (P = 0.259) and tertiary (P = 0.097) follicle numbers were similar. The number of CL was significantly decreased in G3 than in G1 (P = 0.001). The result was similar in terms of cyst formation between G1 and G3 (P = 1.000), (Table 1), (Figures 1a–1c).

When G2 and G3 were compared, primordial (P = 0.165), primary (P = 0.128), tertiary (P = 0.535) follicle, and CL (P = 0.017) numbers were similar. In G2, the number of secondary follicle was significantly decreased compared to G3 (P = 0.001). Cyst formation was significantly higher in G2 than in G3 (P = 0.001), (Table 1), (Figures 1a–1c).

### 3.2. Fibrosis

When G1 and G2 were compared, the fibrosis scores were significantly higher in G2 (P = 0.001), whereas the fibrosis scores were similar when G1 and G3 were compared (P = 0.902). When G2 and G3 were compared, there was a significant increase in the fibrosis score in G2 (P = 0.001), (Table 2), (Figures 1d–1f).

**Table 2:** Comparison of ovarian fibrosis score, apoptosis percentage, and CHRM1 immunoreactivity level among groups.

Groups	Fibrosis score	Apoptosis (%)	CHRM1histoscore
G1 (n = 7)	0.0 (0–0.5)	3 (2–5)	1.2 (0.8–1.8)
G2 (n = 7)	3.0 (2–3)	15 (12–18)	0.4 (0.3–0.6)
G3 (n = 7)	1.0 (0–1)	5 (4–8)	0.9 (0.8–1.8)
P values	G1–G2: 0.001 G1–G3: 0.902 G2–G3: 0.001	G1–G2: 0.001 G1–G3: 0.011 G2–G3: 0.001	G1–G2: 0.001 G1–G3: 0.620 G2–G3: 0.001

Note: Values are presented as median (min–max). G1 = Sham group; G2 = Total salpingectomy group; G3 = Proximal tubal occlusion group; CHRM1 = Cholinergic receptor muscarinic 1

### 3.3. Apoptosis

When G1 and G2 were compared, AI was increased in G2 (P = 0.001). When G1 and G3 were compared, there was a significant increase in AI in G3 (P = 0.011). When G2 and G3 were compared, there was a significant increase in AI in G2 (P = 0.001), (Table 2), (Figures 1g–1i).

### 3.4. MDA levels (nmol/mL)

When MDA values were compared, they were significantly increased in G2 (P = 0.001) and G3 compared to G1 (P = 0.002). Compared with G3, MDA levels were significantly increased in G2 (P = 0.001), (Table 3).

**Table 3:** Comparison of serum AMH and tissue MDA levels among groups.

Groups	AMH (ng/mL)	MDA (nmol/mL)
G1 (n = 7)	6.9 (5.5–8.3)	145 (122–165)
G2 (n = 7)	4.2 (3–5)	687 (415‐766)
G3 (n = 7)	7.3 (5.9–8.1)	263 (162–311)
P values	G1–G2: 0.001 G1–G3: 0.710 G2–G3: 0.001	G1–G2: 0.001 G1–G3: 0.002 G2–G3: 0.001

Note: Values are presented as median (min–max).G1 = Sham group; G 2 = Total salpingectomy group; G 3 = Proximal tubal occlusion groupAMH=

### 3.5. AMH levels (ng/mL)

When G1 and G2 were compared, AMH values were significantly decreased in G2 (P = 0.001), whereas G1 and G3 showed similar values (P = 0.710). When G2 and G3 were compared, G2 showed significantly decreased values (P = 0.001), (Table 3).

### 3.6. CHRM1 immunoreactivity

When G1 and G2 were compared, CHRM1 immunoreactivity was significantly decreased in G2 (P = 0.001). G1 and G3 were similar (P = 0.620). When G2 and G3 were compared, it was significantly decreased in G2 (P = 0.001), (Table 2), (Figures 1j–1l).

## 4. Discussion

This study aimed to compare the effects of PTO performed with bipolar cautery and bilateral TS on ovarian reserve and cholinergic system. In our study, we showed that TS caused ischemic changes in ovarian tissue compared to PTO, causing damage to the ovarian tissue. In the TS group, we found a significant increase in fibrosis, apoptosis scores, and MDA level compared to PTO, and a decrease in ovarian reserve and CHRM1 immunoreactivity. Our findings revealed that, compared with TS, the PTO procedure causes less damage to the local cholinergic function and histopathology of ovaries.

It has been reported that the TS procedure can cause ovarian damage [8–12]. In contrast, when bipolar cautery is used in salpingectomy, the ovarian reserve has been shown to be unaffected [23,25–27]. In our study, we found a significant decrease in the number of secondary follicle and CL in the TS procedure. In addition, our TS group had a significant macroscopic cyst. In our PTO group, no cyst was detected and the follicle numbers were similar to the control group, except for the reduced CL number.

In reproductive ages, the cortex is filled with follicles of different stages and the medulla consists of elastic fibrils, loose connective tissues, blood vessels, lymphatics, and nerve fibers [28]. Given that uterine and tubal lymphatics are very close to each other in the broad ligament, collagen neo-formation is stimulated in blood or lymphatic circulatory disorders [29,30]. By applying TS, we disrupt the ovarian vascular and lymphatic circulation, as well as damage the nerve fibers. When the utero-ovarian vascular anastomosis is disrupted, blood flow deteriorates and hypoxia develops [31]. This explains the increased fibrosis in our TS group. In particular, in rodents, the oxidative damage in oocytes was reported to decrease the fertilization chance by causing mitochondrial damage [32], inhibition of steroidogenic enzymes [33], damage to other proteins involved in the intracellular traffic of cholesterol [34,35], damage to cellular membrane lipids, and DNA damage that may lead to cell death by apoptosis or necrosis [36–38]. In our study tissue MDA levels increased significantly in TS and PTO groups compared to the control group. This shows that both TS and PTO cause oxidative stress in ovarian tissue. However, when we compared the TS and PTO groups, we found that oxidative stress was significantly more severe in the TS group. This shows that TS significantly increases oxidative stress in ovarian tissue compared to PTO. At the same time there was no significant difference in the ovarian reserve and fibrosis score between the control group and the PTO group. This finding shows us that stimulation of oxidative stress after PTO is not as high as TS. Moreover, our findings show that PTO operation damages the ovarian vascular structure significantly less than TS. We thought that salpingectomy can damage other systems in addition to oxidative stress in the ovarian tissue. These findings suggest that explaining the damage in ovarian tissue caused by salpingectomy procedure with only oxidative damage would be inadequate and other mechanisms may play a role. Therefore, we aimed to evaluate the muscarinic receptor activity, which plays a role in vascular functions and ovarian folliculogenesis. Muscarinic receptors are found in vascular endothelial cells. Activation of the muscarinic receptor on vascular endothelial cells causes increased nitric oxide (NO) synthesis that spreads to adjacent vascular smooth muscle cells and causes vasodilatation [39,40]. We thought that TS could affect the ovarian cholinergic system and the functions of neurotransmitters by damaging the vascular and neural structures coming from the broad ligament compared to PTO. We found a relationship between the decrease in CHRM1 activity due to the damage caused by TS in the ovarian tissue as a result of oxidative stress. This determination supports our aim to investigate CHRM1 activity in TS and PTO.

The presence of an intraovarian regulatory system produced by rat and human GCs and containing ACh has been shown. This neurotransmitter may be effective on M 1, M 3, and M 5 muscarinic receptors in GCs of oocytes [41]. GCs also express muscarinic receptors for ACh. Therefore, the presence of an ovarian cholinergic system due to local ACh functions and nonneuronal ACh production has been accepted. Previous studies, however, have described the trophic, growth-promoting effects of ACh on GCs via muscarinic receptors in cattle and human induced ovarian cells. Increases in intracellular Ca 2 + levels, activation of ion channels, and disruption of gap junction communication are among the results of the decrease in cholinergic activity [20,42]. Acetylcholinesterase (AChE) identified in mRNA and protein level is localized at the endocrine and vascular sites of rodent ovaries. It has been found that the enzyme is mainly associated with blood vessels, follicular GCs, and CL. On this basis, it is thought that AChE is associated with rapidly changing endocrine sites and blood vessels of the ovary in adulthood [42]. In a study by Urra et al. [43], it was shown that the inhibition of AChE with huperzine A (Hup A), a specific inhibitor of AChE, for 4 weeks was associated with the increase of the intra ovarian ACh, which results in a strong modification of follicular development and reduction in ovarian cyst formation. ACh can maintain the initial development of follicles up to the antral stage. However, many of these follicles may be exposed to atresia because they are not supported by FSH. In this study, the fact that the initial follicle growth did not change significantly was proven with a similar number of primordial and primary follicle numbers. However, an increase in the number of secondary follicles has been shown. Specifically, an accumulation of small preantral secondary follicles occurred. This suggests that the effects of ACh are focused on supporting the growth of primary follicles [43]. We also did not find any significant difference in primary and primordial follicle numbers in TS and PTO groups. However, macroscopic cyst formation in the TS group, the decrease in the number of secondary follicles, and the decrease in AMH levels, which is regarded as the most reliable test for ovarian reserve and known to be well correlated with the true histological number of ovarian follicles [44], may be a consequence of the decrease in muscarinic activity, thereby affecting ACh. 

This study has a number of limitations. Firstly, it has a very small sample size but we determined the number of rats by using power analysis. Secondly, if we performed ovarian stimulation after two procedures and compared the oocyte counts obtained, our study would be more valuable. Thirdly, the long-term effects of TS and PTO were not studied in our experiment. Short-term effects were studied after 1 month. The reason we prefer the 1 month period is the estrous cycle of the rats, which lasts 4-5 days. In a 28-day follow-up period, a length of 5–7 cycles is considered in rats. The developmental stage of primordial follicle to secondary follicular may take >30 days. The developmental period from secondary stage to gradual ovulation can be 28 ± 2 to 3 days [45]. For these reasons, we planned to investigate the ovaries 4 weeks after TS and PTO in order to be able to evaluate the effects of all follicles in the stages ranging from primordial follicular to ovulation on ovarian reserve after exposure to tubal surgery. Therefore, we examined the early effects of TS and PTO on ovarian tissue. The results may change with longer follow-up period. The fourth limitation is that it is also clear that the experimental results in animal models cannot be fully applied to humans. 

The strength of our study is that it is the first pilot investigation attempting to explain the effects of TS and PTO, which are frequently applied in cases of HX, on the ovarian morphology through oxidative stress and muscarinic receptors. Secondly, ovarian histopathological studies are ethically difficult to perform in the ovary of women of reproductive age. This increases the value of our experimental work.

In conclusion, in the experimental rat model, compared to TS, the PTO procedure does less damage to local cholinergic function and histopathology of the ovaries.

## Conflict of interest

All the authors declare that they have no conflict of interest to disclose.

## Acknowledgement

We thank Professor Dr. Nevin İlhan and Fırat University Biochemistry Clinic for providing biochemical analysis data in this study.
